# A Comparative Study of Ceftazidime–Avibactam and Meropenem-Based Regimens in the Treatment of Carbapenem-Resistant Gram-Negative Bacterial Infections in Intensive Care Units

**DOI:** 10.3390/antibiotics14090863

**Published:** 2025-08-27

**Authors:** Murat Aydın, Nurten Nur Aydın, Mehtap Hülya Aslan, Mithat Kahramanoğlu

**Affiliations:** 1 Department of Infectious Diseases and Clinical Microbiology, Erzurum Regional Training and Research Hospital, 25080 Erzurum, Türkiye; nurtennurkenc@hotmail.com; 2 Department of Microbiology, Erzurum Regional Training and Research Hospital, 25080 Erzurum, Türkiye; hulya_mehtab@hotmail.com; 3 Department of Anesthesiology and Reanimation, Erzurum Regional Training and Research Hospital, 25080 Erzurum, Türkiye; mithatkahramanoglu@hotmail.com

**Keywords:** carbapenem resistance, ceftazidime–avibactam, intensive care unit, combination therapy, mortality

## Abstract

Background: This study aimed to compare mortality rates and treatment efficacy between ceftazidime–avibactam (CAZ/AVI) and meropenem-based combination regimens in critically ill patients with carbapenem-resistant Gram-negative bacteria (CRGNB) infections. Methods: This retrospective study included 135 intensive care unit (ICU) patients diagnosed with CRGNB infections between 2020 and 2024. Patients were categorized on the basis of treatment: CAZ/AVI or alternative combinations that included meropenem with either amikacin or polymyxin. The primary outcomes were 14-day, 30-day, and 90-day all-cause mortality rates. The secondary outcomes included the clinical response on day 14 and the total duration of ICU hospitalization. Results: Among the patients, 74 received CAZ/AVI, whereas 61 were treated with meropenem-based regimens. No significant differences were observed in the baseline characteristics between the groups. There were no statistically significant differences in 14-day (27.0% vs. 31.1%), 30-day (41.9% vs. 47.5%), or 90-day mortality rates (62.2% vs. 65.6%) between the two groups (*p* = 0.738, 0.511, and 0.818, respectively), including within the pneumonia and bloodstream infection subgroups. Clinical success was observed in 64.9% of the CAZ/AVI group and 65.6% of the other group (*p* = 0.931), with comparable ICU lengths of stay (44.0 ± 29.1 vs. 41.5 ± 26.4 days, *p* = 0.974). Multivariate analysis revealed that advanced age, higher Sequential Organ Failure Assessment (SOFA) scores, elevated procalcitonin levels, and prolonged time from culture collection to the initiation of appropriate antibiotic therapy were independent predictors of increased 30-day mortality. Conclusions: CAZ/AVI demonstrated efficacy and mortality outcomes comparable to those of meropenem-based regimens in ICU patients with CRGNB infections. Prompt initiation of appropriate antimicrobial therapy remains critical.

## 1. Introduction

Carbapenem-resistant Gram-negative bacteria (CRGNB) infections have emerged as a significant global public health concern due to limited therapeutic options and high mortality rates, particularly among intensive care unit (ICU) patients [1]. Pathogens such as *Klebsiella pneumoniae* and *Pseudomonas aeruginosa* play crucial roles in the etiology of nosocomial infections [1]. These microorganisms can lead to serious infections, including ventilator-associated pneumonia and bloodstream infections, especially in patients hospitalized in the ICU [2]. This situation presents a significant challenge for clinicians because of the limited availability of effective treatment options. With the increasing prevalence of CRGNBs, investigating novel antimicrobial strategies has become essential for improving patient outcomes.

In recent years, ceftazidime–avibactam (CAZ/AVI), a β-lactam/β-lactamase inhibitor combination, has emerged as a promising agent with potent activity against carbapenem-resistant Enterobacterales and *P. aeruginosa* [3,4]. Nevertheless, carbapenem-based combination therapies, particularly those including polymyxins and aminoglycosides, remain widely used in clinical settings [5,6,7,8]. Several studies have evaluated the efficacy of CAZ/AVI compared with that of conventional agents such as meropenem, polymyxins, and aminoglycosides, with their clinical success rates showing variability [5,6,8]. However, comparative data on the clinical efficacy and mortality outcomes of CAZ/AVI versus meropenem–amikacin and meropenem–polymyxin combinations remain limited, particularly in real-world ICU settings.

This study aimed to compare the effects of CAZ/AVI with those of meropenem–amikacin and meropenem–polymyxin combinations on mortality in patients with CRGNB infections.

## 2. Results

### 2.1. Study Population

A total of 135 patients were included in the study. The mean age was 61.5 ± 15.9 years, and 78 patients (57.8%) were male. The patients were categorized into two groups: 74 (54.8%) received CAZ/AVI, and 61 (45.2%) received alternative antibiotic therapies. No statistically significant differences in demographic or clinical characteristics were observed between the groups (Table 1). The most common comorbidity was hypertension (44.4%), followed by diabetes mellitus (34.8%). Although the mean Charlson Comorbidity Index, APACHE II, and SOFA scores were greater in the CAZ/AVI group than in the alternative treatment group, the differences were not statistically significant.

Pneumonia was the most frequent infection type, identified in 58.1% of patients in the CAZ/AVI group and in 52.5% of those in the alternative treatment group (*p* = 0.511). The most commonly isolated pathogens were *K. pneumoniae* (68.9%) and *P. aeruginosa* (31.1%). Polymicrobial infections were observed in 35.6% of the cases, with *Acinetobacter* spp. being the most frequently co-isolated organism (*n* = 26). Additionally, *Staphylococcus* spp. were identified in nine patients; *Citrobacter* spp., *Enterococcus* spp., and *Stenotrophomonas maltophilia* were identified in eight patients each; and *Escherichia coli* was identified in four patients. Co-infection with *K. pneumoniae* and *P. aeruginosa* was observed in five patients.

The baseline white blood cell counts and C-reactive protein and procalcitonin levels did not differ significantly between the groups. The mean time from culture collection to initiation of antibiotic therapy was 3.0 ± 0.8 days in the CAZ/AVI group and 2.9 ± 0.9 days in the alternative treatment group (*p* = 0.361). The mean duration of treatment was 11.6 ± 3.3 days, with no statistically significant difference between the groups (*p* = 0.105).

### 2.2. Mortality Analysis

The overall mortality rates were 28.9% on day 14, 44.4% on day 30, and 63.7% on day 90. No statistically significant differences were observed in 14-day, 30-day, or 90-day mortality between patients treated with CAZ/AVI and those receiving alternative antibiotic regimens (Table 2). In the pneumonia subgroup, the 14-day, 30-day, and 90-day mortality rates were 37.3%, 54.7%, and 74.7%, respectively. Among patients with bloodstream infections, the 14-day, 30-day, and 90-day mortality rates were 20.0%, 44.0%, and 68.0%, respectively. Clinical success was achieved in 64.9% of patients in the CAZ/AVI group and 65.6% in the alternative treatment group (*p* = 0.931). The mean duration of ICU stay did not differ significantly between the two groups (44.0 ± 29.1 vs. 41.5 ± 26.4 days; *p* = 0.974).

**Figure 1:**
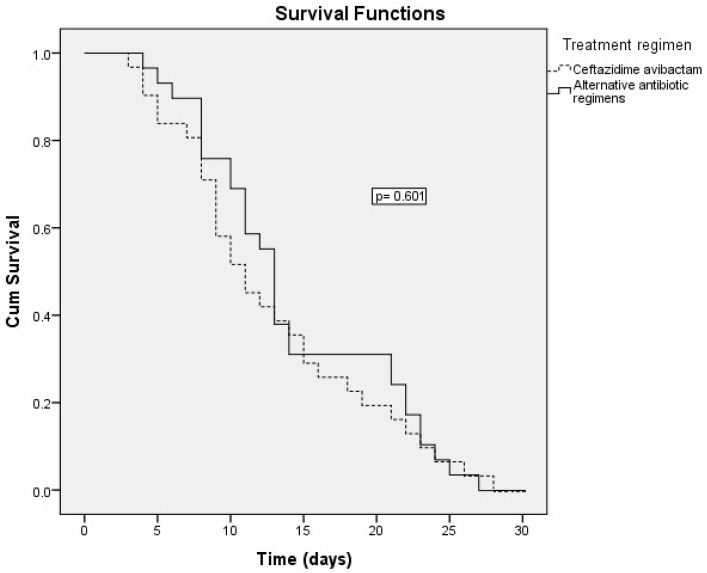
Kaplan–Meier survival curves comparing 30-day survival between patients treated with ceftazidime–avibactam and alternative antibiotic regimens.

Kaplan–Meier survival analysis revealed no significant difference in 30-day survival between patients treated with ceftazidime–avibactam and those receiving alternative antibiotic regimens (*p* = 0.601) (Figure 1).

Analysis of factors associated with mortality revealed that patients who died within 30 days were significantly older than survivors were (64.5 ± 16.1 vs. 59.1 ± 15.4 years; *p* = 0.032). Compared with those of survivors, the Charlson Comorbidity Index, APACHE II, and SOFA scores were significantly greater in patients who died on days 14 and 30. Furthermore, procalcitonin levels were significantly elevated among nonsurvivors (Table 3). Pneumonia was diagnosed in 71.8% of patients who died within the first 14 days and in 68.3% of those who died within 30 days. The presence of pneumonia was significantly associated with both 14-day and 30-day mortality (*p* = 0.026 and *p* = 0.012, respectively). In contrast, no significant associations were observed between mortality and the type of isolated pathogen or the presence of polymicrobial infection (Table 3). Additionally, the time from index culture collection to the initiation of antibiotic therapy was significantly longer in patients who died within 30 days (*p* = 0.003).

In the multivariate logistic regression analysis, advanced age (OR: 1.033; 95% CI: 1.003–1.063; *p* = 0.029), higher SOFA score (OR: 1.164; 95% CI: 1.037–1.306; *p* = 0.010), elevated procalcitonin level (OR: 1.032; 95% CI: 1.010–1.055; *p* = 0.004), and prolonged duration from culture collection to the initiation of appropriate antimicrobial therapy (OR: 2.120; 95% CI: 1.275–3.526; *p* = 0.004) were independently associated with increased 30-day mortality (Table 4).

## 3. Discussion

This study revealed no significant differences in 14- and 30-day mortality rates, clinical success, or length of stay in the ICU between patients treated with CAZ/AVI and those receiving alternative antibiotic regimens (meropenem–amikacin or meropenem–polymyxin combinations) for managing CRGNB infections. Notably, despite higher Charlson Comorbidity Index, APACHE II, and SOFA scores in the CAZ/AVI group, the clinical outcomes were comparable between the two groups. These findings suggest that CAZ/AVI may serve as an effective therapeutic option, even in patients with more severe clinical presentations. The relatively similar outcomes between CAZ/AVI and other regimens suggest implications for determining optimal treatment strategies, particularly in healthcare settings with limited resources.

The efficacy of CAZ/AVI against resistant Gram-negative bacteria has been demonstrated in numerous studies. In particular, its activity against carbapenem-resistant *K. pneumoniae* and *P. aeruginosa* has been well documented in the literature [9,10,11,12]. In a study by Çetinkol et al., the susceptibility rates of carbapenem-resistant *K. pneumoniae* and *P. aeruginosa* strains to CAZ/AVI were reported to be 85.7% and 83.3%, respectively, among 122 CRGNB isolates [13]. Similarly, in a previous study, 88.9% of 352 *K. pneumoniae* strains and 84.5% of 155 *P. aeruginosa* strains isolated from ICUs were susceptible to CAZ/AVI [14]. These findings support the potential role of CAZ/AVI as a promising therapeutic option for infections caused by multidrug-resistant Gram-negative pathogens. Moreover, a meta-analysis reported a significantly lower 30-day mortality rate associated with CAZ/AVI than with other treatment regimens, including colistin-based therapies [12]. In another analysis involving six cohort studies, CAZ/AVI was associated with reduced 30-day mortality in patients with carbapenemase-producing *K. pneumoniae* infections compared with those receiving alternative antibiotics [15]. A retrospective study also revealed a lower 30-day mortality rate in the CAZ/AVI-treated group (27.7%) than in the polymyxin B group (46.7%) [16]. Conversely, a recent study comparing CAZ/AVI with a combination of fosfomycin and meropenem revealed no statistically significant difference in 14- and 30-day mortality rates [17]. Similarly, our study revealed no significant differences in 14-, 30-, or 90-day mortality rates between CAZ/AVI and alternative antibiotic regimens. This may be explained by the fact that, unlike many studies in the literature, our study evaluated meropenem-based combination therapies instead of colistin or polymyxin monotherapies. Indeed, a recent study reported similar mortality rates between patients treated with CAZ/AVI and those receiving a combination of meropenem and fosfomycin [17]. These observations underscore the need for further investigation into the clinical effectiveness of carbapenem-containing combination therapies.

In our mortality analysis, advanced age and higher Charlson Comorbidity Index, APACHE II, and SOFA scores were significantly associated with increased mortality. These findings suggest that serious comorbidities and the severity of disease are key factors influencing mortality. With advancing age, immune system function tends to decline, and the burden of comorbid conditions increases, both of which may compromise the host’s ability to control infections. A systematic review identified older age, comorbidities, and elevated severity scores—such as APACHE II and SOFA scores—as significant risk factors for mortality in patients with carbapenem-resistant Enterobacteriaceae infections [18]. Similarly, a multicenter study involving 339 patients with CRGNB infections reported that higher Charlson Comorbidity Index, APACHE II, and SOFA scores were associated with increased mortality [19]. These findings underscore the importance of comprehensive clinical risk assessment in patients with CRGNB infections.

In our study, pneumonia was the most common type of infection diagnosed in patients, and its presence was significantly associated with mortality on the 14th and 30th days. This suggests that the higher mortality was attributable to the infection site (pneumonia) rather than to the specific pathogen, as *K. pneumoniae* and *P. aeruginosa*—although frequently isolated in pneumonia cases—did not individually show a significant association with mortality in our cohort. This finding highlights pneumonia as an important prognostic marker, particularly for patients in the ICU. Pneumonia plays a crucial role in determining the clinical course of intensive care unit (ICU) patients because of its impact on the respiratory system and its rapid induction of the immune response. Previous studies have reported significantly high mortality rates, particularly in patients with ventilator-associated pneumonia [2,20]. In addition, ventilator-associated pneumonia has been shown to prolong ventilator use, increase the risk of sepsis, and be associated with the presence of multidrug-resistant microorganisms [21,22]. Current findings suggest that pneumonia significantly adversely affects disease progression, especially in critically ill patients, and increases the risk of mortality.

In our study, we found that procalcitonin levels, among the biochemical markers evaluated at the time of diagnosis, were significantly associated with mortality. Procalcitonin has emerged as a crucial biomarker in clinical conditions characterized by a pronounced systemic inflammatory response, such as bacterial infections and sepsis. In addition to reflecting the severity of infection and the extent of systemic inflammation, elevated PCT levels have been shown in previous studies to be correlated with organ dysfunction and an unfavorable clinical course [23]. Our findings suggest that PCT may serve as a valuable biomarker for predicting patient prognosis. Monitoring procalcitonin levels, particularly in ICU patients, is considered to aid in clinical decision-making in optimizing treatment management.

In CRGNB infections, delays in administering appropriate antibiotic therapy significantly impact patient mortality. A previous study demonstrated that longer intervals between culture collection and the initiation of antibiotic therapy are associated with a significant increase in 30-day mortality rates [24]. In our study, the analysis of 30-day mortality revealed that the time from the index culture to the initiation of antibiotic treatment was significantly longer in patients who died (3.2 ± 0.9 days) than in those who survived (2.7 ± 0.7 days). This finding further emphasizes the critical importance of promptly initiating empirical treatment, particularly in severe infections caused by resistant pathogens. Early and appropriate antibiotic therapy can improve patient prognosis by preventing the systemic spread of infection and the onset of sepsis. Therefore, diagnostic and therapeutic strategies should be employed to minimize delays, particularly in high-risk patients.

This study has several limitations. First, it has a retrospective design, which may limit the generalizability of the results, as it was conducted in a single center. Large multicenter prospective studies are needed to confirm our findings. Second, pharmacokinetic and pharmacodynamic variables were not assessed in detail in our study. The absence of serum antibiotic levels may have hindered a comprehensive evaluation of their potential impact on treatment efficacy. Additionally, although the comorbidities and clinical conditions of patients who could influence mortality rates were assessed, potential confounding factors could not be entirely excluded. Moreover, the genotypic characteristics of pathogens, particularly the types of carbapenemase enzymes, were not examined in our study, and the failure to identify resistance mechanisms at the molecular level is considered a significant limitation. Another potential limitation is the requirement to obtain culture and antibiotic susceptibility results before initiating CAZ/AVI treatment in our country. This may have delayed the start of treatment due to the inability to begin empirical therapy, which could have affected the results. Furthermore, the recruitment period (2020–2024) overlapped with the COVID-19 pandemic. During this period, both treatment regimens were primarily prescribed based on physicians’ clinical judgment rather than the year of admission. Although we did not conduct a formal year-by-year analysis, we found no obvious imbalance in treatment distribution across different years. Despite these limitations, our study provides valuable data on mortality rates in patients with CRGNB infections and makes an important contribution to the literature in this field.

## 4. Materials and Methods

This retrospective observational study was conducted on patients hospitalized in the ICU of a tertiary care hospital from 1 January 2020, to 31 December 2024. The study included adult patients diagnosed with infections caused by CRGNBs during hospitalization and treated with CAZ/AVI or other antibiotic regimens, including meropenem–polymyxin combinations or meropenem–amikacin combinations.

### 4.1. Data Collection and Definitions

The demographic characteristics, comorbidities, and infection types of the patients included in the study were recorded. The causative microorganisms, biochemical and hematological parameters, and clinical disease scores—including the Charlson Comorbidity Index, Acute Physiology and Chronic Health Evaluation II (APACHE II), and Sequential Organ Failure Assessment (SOFA) scores—were evaluated. Antibiotic treatment regimens, treatment duration, and the time from index culture to antibiotic initiation were also analyzed. Patient outcomes were evaluated in terms of 14-day, 30-day, and 90-day mortality. All the data were retrospectively collected from patient records and the hospital’s electronic medical system.

Bloodstream infection was defined as the isolation of at least one pathogen from blood cultures in the presence of compatible clinical signs and symptoms. Pneumonia was diagnosed on the basis of at least two of the following criteria: fever (>38 °C), leukocytosis (>10,000/mm^3^) or leukopenia (<4000/mm^3^), purulent sputum, impaired oxygenation, and new or progressive infiltration on chest radiography. Ventilator-associated pneumonia was defined as pneumonia that developed at least 48 h after the initiation of mechanical ventilation. Urinary tract infection was defined as bacterial growth of ≥10^5^ CFU/mL in urine culture accompanied by pyuria; in our cohort, all patients (including those with urinary catheters) met this threshold. Intra-abdominal infection was diagnosed on the basis of the growth of pathogens in relevant samples, supported by clinical, radiological, and laboratory findings.

The SOFA score was calculated on the day of infection diagnosis to assess organ dysfunction. This score incorporates evaluations of respiratory, cardiovascular, hepatic, coagulation, renal, and central nervous system functions [25]. The APACHE II score, a prognostic tool integrating physiological parameters, age, and chronic disease status, was also calculated on the day of diagnosis [26]. Immunosuppression status was determined by evaluating the underlying disease and medications used by patients. Patients were considered immunosuppressed if they had undergone organ transplantation; had active malignancy, HIV infection, or primary immunodeficiency syndrome; or were receiving immunosuppressive therapy (e.g., ≥20 mg/day of prednisone or equivalent, chemotherapy, calcineurin inhibitors, or other cytotoxic agents) [27]. Chronic kidney disease was defined as a glomerular filtration rate <60 mL/min/1.73 m^2^ for at least three months or structural or functional abnormalities indicating renal impairment [28]. Polymicrobial infection was defined as the isolation of more than one pathogen from the same clinical sample within a 72 h period. Coagulase-negative staphylococci and *Corynebacterium* species identified in blood cultures were evaluated on the basis of contamination criteria, and isolates with no clinical significance were excluded from the study.

Clinical success was defined as the resolution or significant improvement of signs and symptoms of CRGNB infection within 14 days after the initiation of treatment. It was assessed according to the following criteria: (i) hemodynamic stability (systolic blood pressure >90 mmHg without the need for vasopressor support), (ii) stability or improvement of the SOFA score (at least a 30% decrease if the baseline SOFA score ≥ 3, stability if <3), (iii) normalization of fever if present, and (iv) stability or improvement of the PaO_2_/FiO_2_ ratio. Failure to meet any of these criteria was considered clinical failure.

#### 4.1.1. Inclusion Criteria

(1) Age ≥ 18 years
(2) Isolation of CRGNB from appropriate clinical samples
(3) Receipt of treatment with either CAZ/AVI or alternative antibiotic regimens (meropenem plus polymyxin or meropenem plus amikacin)
(4) Diagnosis of infection confirmed by clinical and laboratory findings

#### 4.1.2. Exclusion Criteria

(1) Patients with colonization who did not meet the diagnostic criteria for infection
(2) Patients who initiated treatment at an external center and for whom complete clinical data were unavailable.

### 4.2. Microbiological Analysis

The bacterial isolates were identified at the species level via the BD Phoenix ID/AST automated system (BD Diagnostic Systems, Sparks, MD, USA). Antimicrobial susceptibility testing for carbapenems (meropenem, imipenem, and ertapenem) and aminoglycosides (amikacin and gentamicin) was also performed via the same system. The in vitro activity of CAZ/AVI (10/4 mg, Oxoid, Basingstoke, UK) was assessed via the Kirby–Bauer disk diffusion method. All the results were evaluated according to the criteria of the European Committee on Antimicrobial Susceptibility Testing (EUCAST) [29].

### 4.3. Treatment Approaches

Patients were categorized into two groups on the basis of the administered treatment: those who received CAZ/AVI and those who received alternative antibiotic regimens. The choice of antibiotic regimens was not guided by predefined criteria but by the treating physicians’ clinical judgment and microbiological data. CAZ/AVI was administered intravenously at a dose of 2.5 g every 8 h. In patients with renal impairment, the dosage was adjusted according to creatinine clearance (CrCl) as follows: 1.25 g IV every 8 h for CrCl 31–50 mL/min; 0.94 g IV every 12 h for CrCl 16–30 mL/min; 0.94 g IV every 24 h for CrCl 6–15 mL/min (with or without hemodialysis [HD], and an additional post-HD dose on dialysis days); and 1.25 g IV every 8 h for patients undergoing continuous renal replacement therapy (CRRT).

In the other antibiotic regimens group, treatment consisted of either meropenem plus polymyxin (colistin or polymyxin B) or meropenem plus amikacin. Meropenem was administered as a 1000 mg intravenous infusion over 30 min every 8 h. For patients with renal impairment, the dose was adjusted according to CrCl: 1 g IV every 12 h for CrCl 26–49 mL/min; 0.5 g IV every 12 h for CrCl 10–25 mL/min; and 0.5 g IV every 24 h post HD for CrCl < 10 mL/min or intermittent HD.

Colistin was given as a 9 million unit (MU) loading dose, followed by maintenance doses of 4.5 MU every 12 h, adjusted for renal function in patients with CrCl. Polymyxin B was administered at a loading dose of 2.5 mg/kg (25,000 units/kg) and maintenance doses of 1.25–1.5 mg/kg (12,500–15,000 units/kg) every 12 h.

Amikacin was administered intravenously at 15 mg/kg/day. For patients with renal impairment, the dosage was adjusted as follows: 7.5 mg/kg every 24 h for CrCl 10–50 mL/min and 7.5 mg/kg every 48 h for CrCl < 10 mL/min.

### 4.4. Statistical Analysis

Statistical analyses were conducted via IBM SPSS Statistics version 23.0 (IBM Corp., Armonk, NY, USA). Descriptive statistics are presented as frequencies (*n*) and percentages (%) for categorical variables and as the means ± standard deviations for numerical variables. The chi-square test was used to compare categorical variables between independent groups. The normal distributions of the numerical variables were evaluated via the Shapiro–Wilk W test and the Kolmogorov‒Smirnov test. Student’s *t* test was applied for normally distributed variables for comparisons between two independent groups, whereas the Mann–Whitney *U* test was used for nonnormally distributed variables. Predictors of 30-day mortality were examined via multivariate logistic regression analyses based on forward stepwise selection of variables with a *p* value of less than 0.05 in univariate analysis, along with an odds ratio and a 95% confidence interval. A *p* value of < 0.05 was considered to indicate statistical significance.

## 5. Conclusions

In conclusion, our study presents important findings on treatment outcomes and mortality-associated risk factors in patients with CRGNB infections. Although no statistically significant differences were observed in 14-, 30-, or 90-day mortality rates between CAZ/AVI and other antibiotic regimens, these results should be confirmed through large-scale, prospective, randomized controlled trials. Future studies are warranted to further evaluate the efficacy of CAZ/AVI in specific patient subgroups and to inform optimal treatment strategies for managing resistant Gram-negative infections.

## Figures and Tables

**Table 1:** Characteristics of patients receiving CAZ/AVI-based and alternative antibiotic regimens.

Variables	CAZ/AVI (*n* = 74)	Alternative Antibiotic Regimens (*n* = 61)	Total (*n* = 135)	*p* Value
Age, years ± SD	62.4 ± 15.3	60.4 ± 16.6	61.5 ± 15.9	0.704
Gender (male)	44 (59.5%)	34 (55.7%)	78 (57.8%)	0.663
Underlying disease				
Hypertension	33 (44.6%)	27 (44.3%)	60 (44.4%)	0.969
Diabetes mellitus	27 (36.5%)	20 (32.8%)	47 (34.8%)	0.789
Cerebrovascular disease, dementia	25 (33.8%)	19 (31.1%)	44 (32.6%)	0.888
COPD	23 (31.1%)	16 (26.2%)	39 (28.9%)	0.669
Cardiovascular disease	22 (29.7%)	13 (21.3%)	35 (25.9%)	0.361
Chronic kidney disease	19 (25.7%)	12 (19.7%)	31 (23.0%)	0.535
Malignancy	15 (20.3%)	14 (23.0%)	29 (21.5%)	0.867
Immunosuppression	13 (17.6%)	15 (24.6%)	28 (20.7%)	0.431
Severity of illness				
CCI ± SD	6.8 ± 3.7	5.7 ± 3.0	6.3 ± 3.4	0.131
APACHE II score ± SD	21.4 ± 6.8	19.4 ± 6.9	20.5 ± 6.9	0.088
SOFA score ± SD	8.2 ± 4.1	7.2 ± 3.3	7.7 ± 3.8	0.129
Type of infection				
Pneumonia	43 (58.1%)	32 (52.5%)	75 (55.6%)	0.511
Bloodstream (including CRBSI)	10 (13.5%)	15 (24.6%)	25 (18.5%)	0.154
Urinary tract	7 (9.5%)	10 (16.4%)	17 (12.6%)	0.343
Intraabdominal	10 (13.5%)	2 (3.3%)	12 (8.9%)	0.076
Other	4 (5.4%)	2 (3.3%)	6 (4.4%)	0.689
Baseline pathogen				
*Klebsiella pneumoniae*	54 (73.0%)	39 (63.9%)	93 (68.9%)	0.346
*Pseudomonas aeruginosa*	20 (27.0%)	22 (36.1%)	42 (31.1%)	0.346
Polymicrobial infection	22 (29.7%)	26 (42.6%)	48 (35.6%)	0.169
White blood cell count (/µL) ± SD	11,624 ± 5617	12,316 ± 4702	11,991 ± 5140	0.335
C-reactive protein (mg/L) ± SD	172.8 ± 73.0	172.1 ± 75.1	172.5 ± 73.7	0.954
Procalcitonin (ng/mL) ± SD	17.2 ± 28.5	13.6 ± 22.8	15.6 ± 26.1	0.363
Time to antibiotic initiation from index culture (days) ± SD	3.0 ± 0.8	2.9 ± 0.9	2.9 ± 0.8	0.361
Duration of antibiotic therapy (days) ± SD	11.3 ± 3.4	12.1 ± 3.2	11.6 ± 3.3	0.105

APACHE: Acute Physiology and Chronic Health Evaluation, CAZ/AVI: ceftazidime avibactam, CCI: Charlson Comorbidity Index, COPD: chronic obstructive pulmonary disease, CRBSI: catheter-related bloodstream infection, SD: standard deviation, SOFA: Sequential Organ Failure Assessment.

**Table 2:** Comparison of mortality rates, clinical success, and ICU stay duration between patients treated with CAZ/AVI and alternative antibiotic regimens.

Variables	CAZ/AVI (*n* = 74)	Alternative Antibiotic Regimens (*n* = 61)	Total (*n* = 135)	*p* Values
14-day mortality	20 (27.0%)	19 (31.1%)	39 (28.9%)	0.738
30-day mortality	31 (41.9%)	29 (47.5%)	60 (44.4%)	0.511
90-day mortality	46 (62.2%)	40 (65.6%)	86 (63.7%)	0.818
14-day mortality in the pneumoniae subgroup	15 (33.3%)	13 (40.6%)	28 (37.3%)	0.789
30-day mortality in the pneumoniae subgroup	23 (51.1%)	18 (56.3%)	41 (54.7%)	0.998
90-day mortality in the pneumoniae subgroup	32 (74.4%)	24 (75.0%)	56 (74.7%)	1.000
14-day mortality in BSI subgroup	1 (10.0%)	4 (26.7%)	5 (20.0%)	0.615
30-day mortality in BSI subgroup	5 (50.0%)	6 (40.0%)	11 (44.0%)	0.697
90-day mortality in BSI subgroup	8 (80.0%)	9 (60.0%)	17 (68.0%)	0.402
Clinical success	48 (64.9%)	40 (65.6%)	88 (65.2%)	0.931
Total duration of ICU hospitalization (days)	44.0 ± 29.1	41.5 ± 26.4	42.9 ± 27.9	0.974

BSI: bloodstream infection, CAZ/AVI: ceftazidime avibactam, ICU: intensive care unit.

**Table 3:** Characteristics of patients and univariate analysis of factors associated with 14-day and 30-day mortality.

Variables	14-Day Mortality (*n* = 39)	14-Day Survival (*n* = 96)	*p* Value	30-Day Mortality (*n* = 60)	30-Day Survival (*n* = 75)	*p* Value
Age, years ± SD	63.5 ± 17.4	60.7 ± 15.2	0.230	64.5 ± 16.1	59.1 ± 15.4	0.032
Gender (male)	26 (66.7%)	52 (54.2%)	0.254	37 (61.7%)	41 (54.7%)	0.520
Underlying disease						
Hypertension	20 (51.3%)	40 (41.7%)	0.408	29 (48.3%)	31 (41.3%)	0.416
Diabetes mellitus	20 (51.3%)	27 (28.1%)	0.018	26 (43.3%)	21 (28.0%)	0.094
COPD	16 (41.0%)	23 (24.0%)	0.076	21 (35.0%)	18 (24.0%)	0.226
Chronic kidney disease	10 (25.6%)	21 (21.9%)	0.806	14 (23.3%)	17 (22.7%)	1.000
Immunosuppressive condition	10 (25.6%)	18 (18.8%)	0.509	14 (23.3%)	14 (18.7%)	0.652
Cardiovascular disease	13 (33.3%)	22 (22.9%)	0.301	19 (31.7%)	16 (21.3%)	0.245
Cerebrovascular disease, dementia	12 (30.8%)	32 (33.3%)	0.932	22 (36.7%)	22 (29.3%)	0.472
Malignancy	10 (25.6%)	19 (19.8%)	0.604	15 (25.0%)	14 (18.7%)	0.497
Severity of illness						
CCI ± SD	7.8 ± 3.6	5.7 ± 3.2	0.001	7.3 ± 3.4	5.5 ± 3.2	0.003
APACHE II score ± SD	24.7 ± 6.2	18.8 ± 6.4	<0.001	23.4 ± 6.0	18.3 ± 6.7	<0.001
SOFA score ± SD	10.4 ± 3.4	6.7 ± 3.4	<0.001	9.3 ± 3.6	6.5 ± 3.5	<0.001
Type of infection						
Pneumonia	28 (71.8%)	47 (49.0%)	0.026	41 (68.3%)	34 (45.3%)	0.012
Bloodstream infection (including CRBSI)	5 (12.8%)	20 (20.8%)	0.400	11 (18.3%)	14 (18.7%)	1.000
Urinary tract	3 (7.7%)	14 (14.6%)	0.393	11 (14.7%)	6 (10.0%)	0.582
Intraabdominal	-	12 (12.5%)	0.019	1 (1.7%)	11 (14.7%)	0.020
Other	2 (5.1%)	4 (4.2%)	1.000	2 (3.3%)	4 (5.3%)	0.692
Baseline pathogen						
*Klebsiella pneumoniae*	22 (56.4%)	71 (74.0%)	0.073	36 (60.0%)	57 (76.0%)	0.071
*Pseudomonas aeruginosa*	17 (43.6%)	25 (26.0%)	0.073	24 (40.0%)	18 (24.0%)	0.071
*Escherichia coli*	-	4 (4.2%)	0.324	-	4 (5.3%)	0.129
Polymicrobial infection	12 (30.8%)	36 (37.5%)	0.588	17 (28.3%)	31 (41.3%)	0.165
White blood cell count (/µL) ± SD	12,515 ± 5810	11,732 ± 4795	0.448	12,664 ± 5568	11,281 ± 4590	0.164
C-reactive protein (mg/L) ± SD	187.4 ± 80.8	166.4 ± 70.1	0.135	183.7 ± 77.2	163.5 ± 69.9	0.114
Procalcitonin (ng/mL) ± SD	29.4 ± 34.9	9.7 ± 18.6	<0.001	24.4 ± 33.1	8.1 ± 14.7	<0.001
Duration (in days) from culture to antibiotherapy ± SD	3.2 ± 0.9	2.9 ± 0.8	0.054	3.2 ± 0.9	2.7 ± 0.7	0.003

APACHE: Acute Physiology and Chronic Health Evaluation, CCI: Charlson Comorbidity Index, COPD: chronic obstructive pulmonary disease, CRBSI: catheter-related bloodstream infection, SD: standard deviation, SOFA: Sequential Organ Failure Assessment.

**Table 4:** Multivariate logistic regression analysis of risk factors for 30-day mortality.

Variables	B	S.E.	Wald	OR (95% CI)	*p* Value
Age	0.032	0.015	4.745	1.033 (1.003–1.063)	0.029
SOFA score	0.152	0.059	6.664	1.164 (1.037–1.306)	0.010
Procalcitonin level	0.032	0.011	8.222	1.032 (1.010–1.055)	0.004
Duration (in days) from culture to antibiotherapy	0.751	0.259	8.385	2.120 (1.275–3.526)	0.004

CI: confidence intervals, OR: odds ratios, SOFA: Sequential Organ Failure Assessment.

## Data Availability

The datasets used and/or analyzed during the current study are available from the corresponding author upon reasonable request.
